# Non-contact optical characterization of negative pressure in hydrogel voids and microchannels

**DOI:** 10.1007/s12200-022-00016-5

**Published:** 2022-04-14

**Authors:** Shihao Xu, Xiaowei Liu, Zehua Yu, Kang Liu

**Affiliations:** 1 MOE Key Laboratory of Hydrodynamic Transients, School of Power and Mechanical Engineering, Wuhan University, Wuhan, 430072 China; 2 Wuhan National Laboratory for Optoelectronics, Huazhong University of Science and Technology, Wuhan, 430074 China

**Keywords:** Hydrogel, Negative pressure, Non-contact, Optical

## Abstract

Negative pressure in water under tension, as a thermodynamic non-equilibrium state, has facilitated the emergence of innovative technologies on microfluidics, desalination, and thermal management. However, the lack of a simple and accurate method to measure negative pressure hinders further in-depth understanding of the properties of water in such a state. In this work, we propose a non-contact optical method to quantify the negative pressure in micron-sized water voids of a hydrogel film based on the microscale mechanical deformation of the hydrogel itself. We tested three groups of hydrogel samples with different negative pressure inside, and the obtained results fit well with the theoretical prediction. Furthermore, we demonstrated that this method can characterize the distribution of negative pressure, and can thus provide the possibility of investigation of the flow behavior of water in negative pressure. These results prove this technique to be a promising approach to characterization of water under tension and for investigation of its properties under negative pressure.

**Graphical Abstract:**

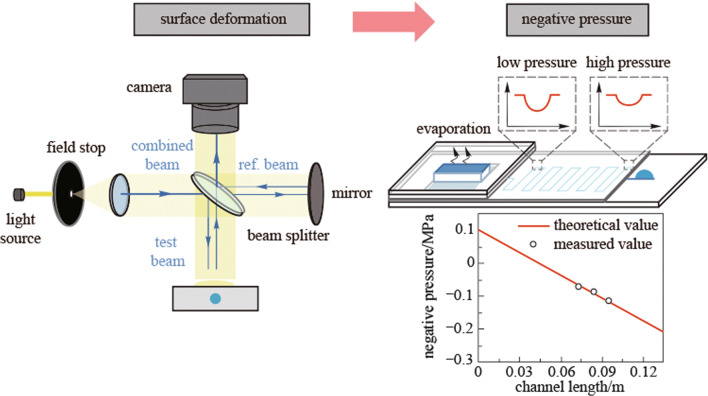

**Supplementary Information:**

The online version contains supplementary material available at 10.1007/s12200-022-00016-5.

## Introduction

Due to cohesive forces, water is able to withstand a stretching force, or tension. Under extreme tension, the hydrostatic pressure of water would exhibit as absolute negative [1], which is a unique thermodynamic non-equilibrium state in the phase diagram of water. Negative pressure of stretched water has been widely observed by botanists in the xylem of trees [2–5]. The negative pressure helps to lift liquid water along the xylem to treetops higher than 10 m. Inspired by this phenomenon, a series of ingenious artificial devices have been developed to implement advanced heat and mass transfer applications. For example, an on-chip synthetic tree device has been developed to generate the pressure of − 1.0 MPa and to passively drive a hydraulic system with large flow resistance [6]. Synthetic mangrove was designed to produce a high negative pressure of − 40 MPa for reverse-osmosis desalination [7]. Negative pressure in nanoporous membranes has also been demonstrated to dissipate unusual high interfacial heat fluxes in thin-film evaporation [8, 9].

Despite great potential in a wide range of applications [10–15], limited measurement methods for negative pressure become the bottleneck that hinders the further investigation and utilization of negative pressure. In most of previous researches, the value of negative pressure was obtained through theoretical derivation from thermodynamic equilibrium [6, 16–18]. However, the theoretical framework relating to the negative pressure domain is still unclear [19]. Possible discrepancy between measurement and theoretical derivation might exist. Some researchers have also tried to characterize the negative pressure in water by hydrophone [20], microtensiometer membrane [21] or localized density characterization [22]. But all these approaches employ sensors that contact the metastable stretched water, and may affect the microenvironment in which the negative pressure forms. Moreover, fabrication of the measurement components is usually complicated and this limits its further application.

In this work, we present a non-contact and convenient optical method to directly measure the negative pressure in micron-sized water voids of a hydrogel film. With the help of 3D optical surface profiler, we observed that ultrahigh negative pressure in the hydrogel voids induced microscale deformation on the hydrogel surface in the form of surface pit. Moreover, we found that the depth of these surface pits induced by negative pressure in the hydrogel voids could reflect the value of negative pressure. Through COMSOL simulation, we established the relationship between the value of negative pressure, depth of the surface pit and geometry parameters of the hydrogel voids. Based on the simulation results, we derived the value of negative pressure inside the hydrogel voids, which agree well with the theoretical calculation. Furthermore, we employed this method to map the negative pressure in a microchannel. Our results open up a new way to characterize the negative pressures and properties of stretched water that operates not only in spherical voids but also in dynamic flow in microchannels.

## Results and discussion

**Fig. 1:**
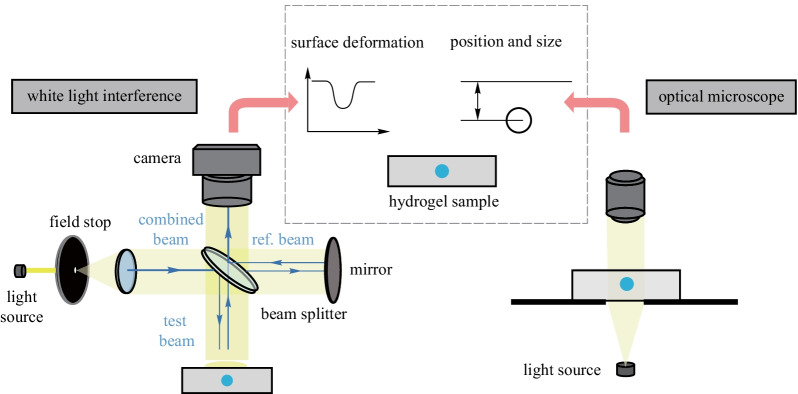
Schematic of the non-contact optical characterization of negative pressure in hydrogels. Surface deformation induced by the negative pressure inside the hydrogel void is characterized by a 3D optical surface profiler. The position and size of the corresponding hydrogel void are measured using an optical microscope

Figure 1 illustrates the working principle of the non-contact optical characterization approach. Negative pressure was generated inside the voids of a hydrogel film by controlling the steady-state ambient humidity [6, 23]. The negative pressure would lead to remarkable micro-scale deformation of the hydrogel if the void were to be close to the surface. The extent of the deformation reflects the value of the negative pressure. Thus, we can derive the negative pressure value by characterizing the surface deformation. Here the surface deformation was first measured by a 3D optical surface profiler (ZYGO NewView™ 9000) through white light interference (Fig. 1 left). Then the positions and sizes of corresponding hydrogel voids were characterized by an optical microscope (Fig. 1 right). Finally, through mechanical inversion calculation, the value of negative pressure could be derived from the surface deformation and position and diameter of the voids.

**Fig. 2:**
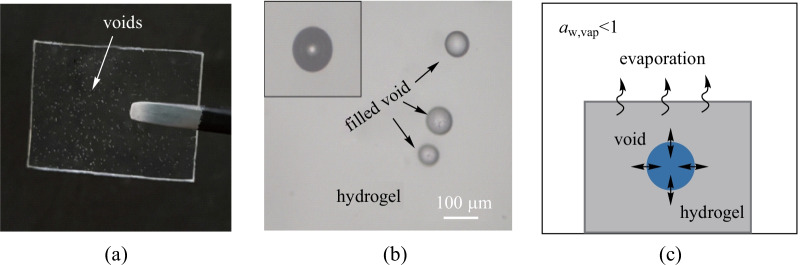
Generation of negative pressure inside the hydrogel voids. **a** Photograph of the hydrogel film with water filled voids. **b** Optical micrograph of hydrogel voids filled with water and after cavitation (left inset). **c** Schematic of the setup that generates negative pressure in hydrogel voids through vapor–liquid equilibrium

To verify the feasibility of the method, we produced and measured negative pressure in hydrogel voids. The employed hydrogel is poly (hydroxyethyl methacrylate) (pHEMA), which has previously been proved to generate negative pressure as high as − 22 MPa in the voids within the hydrogel [6]. Micro spherical voids inside the pHEMA hydrogel were generated with the aid of vortex mixer in the fabrication process. Details can be found in Additional file 1: Method. The voids were filled with water by soaking the hydrogel in boiling deionized water for 48 h (Additional file 2: Fig. S1). The fabricated hydrogel film is as shown in Fig. 2a. Figure 2b shows a microscopic image of the voids.

To generate negative pressure inside the voids, the hydrogel film was placed in a sealed petri dish, in which the environmental humidity was carefully controlled by different types of saturated salt solution (Additional file 3: Fig. S2). When the controlled humidity was lower than 100%, water inside the hydrogel film could be expected to gradually evaporate. In an equilibrium state, the remaining water in the voids then becomes tensioned before cavitation occurs. The pressure of the water reduces accordingly. If the hydrogel is dehydrated, cavitation occurs in the void and breaks the negative pressure. Here it should be noted that cavitation in the voids could be easily distinguished from intact water voids through microscopic image, as shown in Fig. 2b inset. Theoretically, the equilibrium pressure of water in the voids under different ambient humidities could be calculated through the Kelvin–Laplace equation [6]:

1 $$P_{{\text{w}}} = P_{{{\text{atm}}}} + \frac{RT}{{V_{{\text{w}}} }}\ln \left( {\frac{{P_{{{\text{vap}}}} }}{{P_{{{\text{vap}},{\text{sat}}}} }}} \right),$$

where *P*_w_ is the pressure of water in the voids, *P*_atm_ is the pressure of ambient environment, *R* is the gas constant (8.314 J/(mol·K)), *T* is the Kelvin temperature (294 K), *V*_w_ is the molar volume of liquid water (1.8⨯10^–^^5^ m^3^/mol), *P*_vap_ and *P*_vap,sat_ are the ambient vapor pressure and saturated vapor pressure of water, respectively.  *P*_vap_/*P*_vap,sat_ is the relative humidity.

**Fig. 3:**
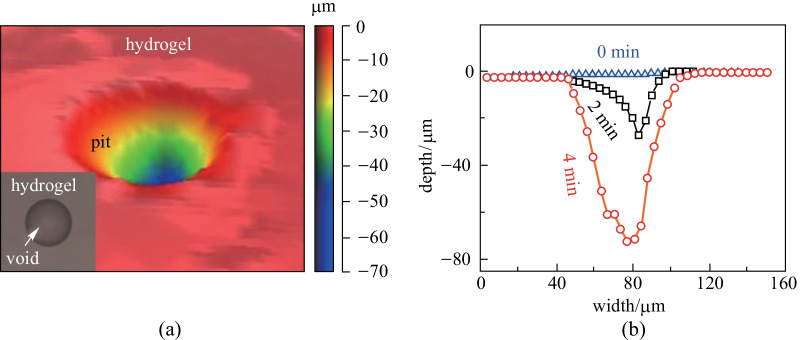
Dynamic formation of a pit on the hydrogel surface. **a** Surface characterization by a 3D optical surface profiler above a selected void. Inset image shows that the void is full filled by water during observation. **b** Dynamic change of surface pit within 4 min

According to Eq. (1), the equilibrium pressure inside the voids becomes negative when the ambient humidity is lower than 99.93%. With this negative pressure, the hydrogel around the void would deform due to the pressure difference between the voids and the ambient environment. To be more specific, such remarkable deformation could be observed on the hydrogel surface especially when the void is close to the hydrogel surface. Figure 3a shows the surface morphology of a hydrogel film after being exposed under the humidity of 40%RH and room temperature of 20 °C for 48 h. Above a micro void, a sharp pit can be observed. The depth is about 75 µm. In a control experiment without water-filled void, no pit was observed on the hydrogel (Additional file 4: Fig. S3b). To further confirm that the pit was generated by the induced negative pressure during evaporation, we monitored the dynamic formation of a surface pit as shown in Fig. 3b and Additional file 4: Fig. S3d. The surface of hydrogel was flat at the initial stage (blue line). As exposure time increased, a small pit formed on the surface at 2 min (black line). Then the depth of the pit kept growing until 4 min (red line). Finally, the geometry of pit remained almost unchanged. These results showed that formation of the pit originated from the evaporation process. During the evaporation, water inside the voids became tensioned, reducing the static pressure and leading to formation of the pit. Therefore, it is theoretically possible to derive the negative pressure in voids by characterizing the hydrogel deformation above the voids.

To test the idea, we prepared a collection of hydrogel samples and maintained the voids at different negative pressures of − 3.3, − 6.9, and − 22 MPa. To achieve these negative pressures, the samples were placed in environments with the ambient humidities of 97.5%, 95%, and 85%, respectively, using different saturated salt solutions (Additional file 5: Table S1). The ambient temperature was 21 °C. Samples were exposed in the given environment for 7 days to ensure complete equilibrium. After that, the geometry of the selected voids, including diameter and distance away from the surface, were characterized by a microscope (Additional file 6: Fig. S4). During the observation, the surface of the hydrogel was coated with an ultrathin polyethylene film to avoid unexpected evaporation. Then, surface deformation above the selected voids was characterized by a 3D optical surface profiler. Table 1 shows the depth of the pits for voids with different sizes and at different negative pressures. It can be clearly seen that, depth of pit is apparently larger at higher negative pressure, with voids of larger diameter and closer to the surface.

**Table 1:** Geometry information of the voids at different negative pressures

Theoretical pressure/MPa	Void diameter/μm	Void depth/μm	Pit depth/μm
− 3.3	85.7	62.7	10.2
	78.2	59.7	7.7
	102.0	91.4	4.2
− 6.9	42.6	48.3	2.8
	53.8	53.9	5.0
	57.0	73.3	4.2
− 22	102.1	81.3	36.6
	130.8	106.0	37.0
	107.4	78.6	31.8

**Fig. 4:**
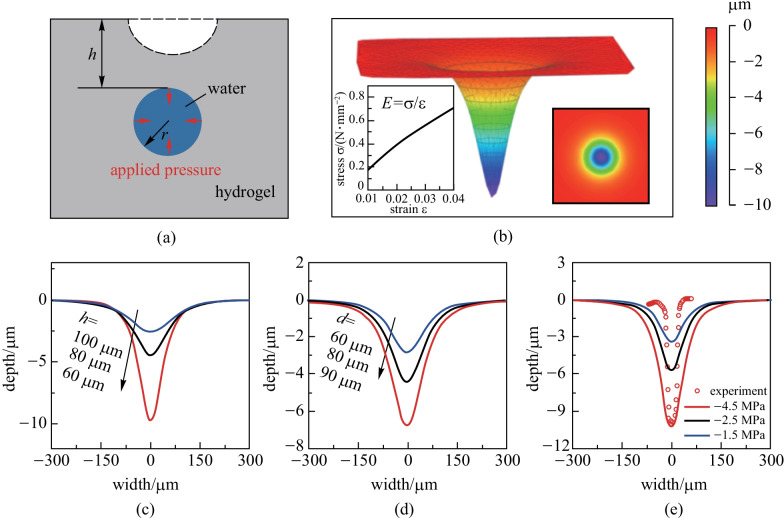
Inversion simulation of the negative pressure inside the voids. **a** Schematic of the simulation model. **b** Simulated pit on the hydrogel surface over a void with the depth of 62.7 μm and pore size of 85.7 μm. Left inset shows the measured variation of stress with strain in the hydrogel. Right inset is vertical view of the simulated pit. **c** and **d** Variation of the pit with pores at different positions and of different diameters. **e** Surface pit depth with different applied pressure on hydrogel void surface

With the geometry information of the voids and the deformations above them, we tried to derive the practical negative pressure inside the voids with inversion simulation as schematically shown in Fig. 4a. A mechanics model of the hydrogel was established with COMSOL Multiphysics. Density of the hydrogel was taken as 1150 kg/m^3^ according experimental measurements. Young’s modulus was taken as 20 MPa (Additional file 7: Fig. S5). Poisson’s ratio was 0.45. Here the stress exhibited a linear dependence on the strain rate before the breakdown (Fig. 4b inset), thus, the hydrogel was taken as linear elastic in simulation. Size and position of the voids were set also according to experimental results. Negative pressure inside the voids was reproduced by applying equivalent centripetal pressure on the outer surface of the void.

Figure 4b shows the simulated surface deformation above the void. The shape of the formed pit is similar to the experimental observation in Fig. 3. Through the simulation results, Fig. 4c suggests that the deformation of the hydrogel surface increases as the depth of the void from the surface (*h*) decreases. When fixing the depth of the void and gradually increasing its size, the deformation also increases (Fig. 4d). With a certain depth and pore size measured in experiments, we could derive the negative pressure by fitting the shape of the surface pit which is obtained by experiments. Figure 4e shows the fitting process with the depth of 62.7 μm and pore size of 85.7 μm in diameter. We gradually changed the applied pressure until the simulated depth fitted well with the experiment results. In this case, the applied pressure was finally derived as 4.5 MPa. Here it is worth noting that the width of the observed split in experiment is narrower than the simulated split, which might be caused by the variation of mechanical strength with the water content (demonstrated in Additional file 8: Fig. S6). Further analysis also shows that such a discrepancy in width would not affect the derivation value of negative pressure.

**Fig. 5:**
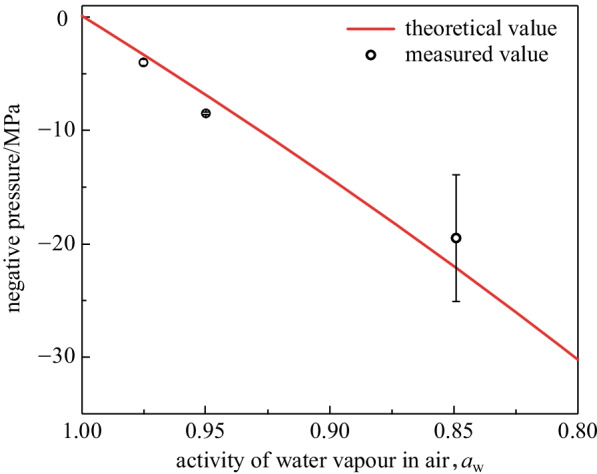
Calculated negative pressure inside the voids under different ambient conditions. Theoretical prediction was also plotted for comparison. The error bar represents standard derivation for three measurements in different voids

With the simulation, we calculated the negative pressures in all the voids as in Table 1. As shown in Fig. 5, the obtained negative pressure values are consistent with the theoretical prediction. It implies that the non-contact optical measurement method could provide an accurate characterization of negative pressure inside the hydrogel voids. Negative pressure value beyond − 25 MPa is not observed in this work due to cavitation, which is also consistent with previous researches.

**Fig. 6:**
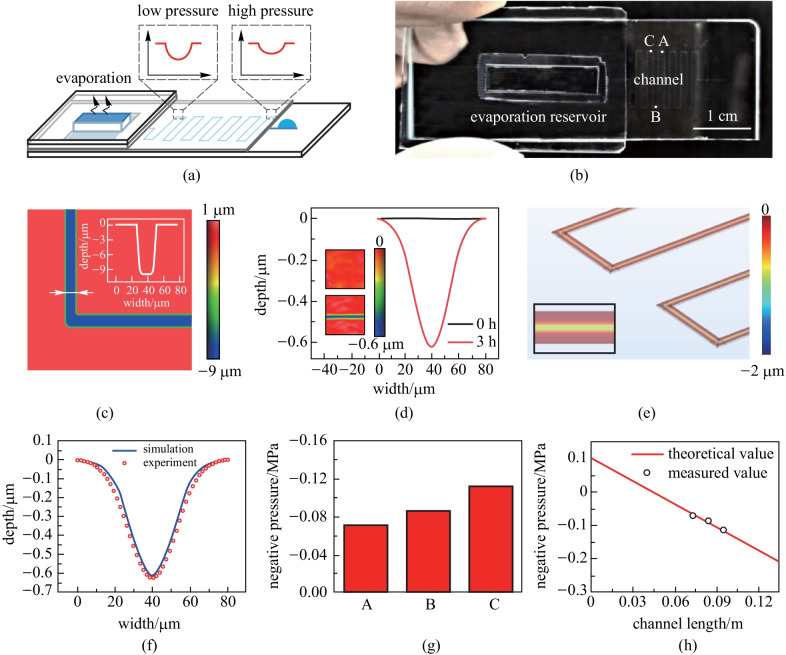
Negative pressure measurement of the fluidic flow. **a** Schematic of the microfluidic chip. **b** Photograph of the chip. **c** Characterization of the microchannel. The inset graph shows the width and depth of the channel. **d** Characterization of the film surface above the channel before and after the evaporation. **e** Image of the model in simulation software. The depth of the surface depression is shown in different colors. Inset graph shows an enlarged view of the deformation on the channel. Concave can be observed on the surface as we observed in experiments. **f** Fitting of the shape of the concave. **g** Measured values of negative pressure at the point of A, B and C. **h** Comparison of the measured pressures and the theoretical values

Results above demonstrate suitability of the method for measurement of the negative pressure inside a void with the size of several tens to hundreds of micrometers. However, according to the mechanism, such a method might be expanded to characterize one-dimensional or two-dimensional distribution of negative pressure for characterizing fluidic flow in negative pressure regions. To achieve this function, we designed a microfluidic chip which could drive the motion of the liquid at negative pressure (Fig. 6a). A glass substrate with microchannel was covered with 20 μm thick PDMS (polydimethylsiloxanes) film. One end of the microchannel was exposed to fill the microchannel with water. The other end of the microchannel connected to the evaporation reservoir which was covered by a piece of pHEMA hydrogel. To make the chip airtight and stable, the hydrogel was embedded in a piece of glass (Additional file 9: Fig. S7). During evaporation, the end of pHEMA hydrogel could generate negative pressure and drive water flowing along the channel, resulting in a pressure drop. Depressions induced by such a pressure drop can be observed on the PDMS film. Figure 6b shows the photograph of the chip. The microchannel had a length of 13.4 cm, width of 25 μm and depth of 10 μm (Fig. 6c).

After filling the channel with water, we exposed the hydrogel to the ambient environment (25 °C and 70%RH). A continuous water supply was also provided from one end of the channel. Before the evaporation occurs, the surface of the PDMS was flat (Fig. 6d). After 3 h evaporation, concave features formed. The water evaporation rate was 0.14 mg/min. More importantly, we checked different points along the channel and found the concave feature became deeper along the flow direction (Additional file 10: Fig. S8). The results showed the potential of the method in mapping the pressure distribution along the channel. To further characterize the exact pressure in these places through the shape of the concave surface, we established a mechanical model of the channel as shown in Fig. 6e. The model consisted of a rigid substrate, a deformable film, and a linear load that increased along the flow on the thin film as a boundary condition. The Young’s modulus of the film was set as 4 MPa, and the Poisson’s ratio was set as 0.475 from an average value of PDMS thin film [24]. By adjusting the boundary load on the film, we fitted the depth of the concave surface to the experiment results and obtained the value of negative pressure (Fig. 6f).

Figure 6g shows the measured pressure at the points A, B, C (marked in Fig. 6b), which are − 0.070, − 0.086, and − 0.112 MPa, respectively (Fig. 6g). As the properties of water in negative pressure could be different from that of bulk water, here we also calculated the theoretical negative pressures at these points with the flow resistances in the microchannel as shown in Fig. 6h. The physical properties of the water were taken to be the same as that of bulk water. The theoretical results are consistent with the experiment results, indicating the accuracy of our method. Also, the results prove that the tensioning or negative pressure of water does not induce significant difference of property of water in the low-negative-pressure region. However, in high-negative-pressure region, in which the tensioned state of water molecules is more intense, the flow behavior may become different [25–27].

## Conclusion

In summary, we reported that negative pressure inside hydrogel voids would induce mechanical deformation of the hydrogel, and lead to formation of surface pits if the voids are close to the surface. Utilizing this phenomenon, we propose a non-contact optical method to quantify the negative pressure in micron-sized water voids of a hydrogel film. Surface pits were characterized by a 3D optical surface profiler, and position of the voids were obtained through focus control of a microscope. Finally, negative pressure was determined by mechanical inversion calculation. Using a pHEMA hydrogel film, we demonstrated the accuracy of the method, and the measured negative values were consistent with the theoretical values. Furthermore, we demonstrated that this method also works to characterize the distribution of negative pressure in a micro channel. These results provide a new and convenient technique to experimentally characterize negative pressure and facilitate the further investigation of thermodynamic or fluidic properties of water under negative pressure.

## Supplementary Information

**Additional file 1.** Supplementary Method. Experimental details.

**Additional file 2. Supplementary Fig. S1.** Fabrication of hydrogels with voids.

**Additional file 3. Supplementary Fig. S2.** Schematic of the method to generate negative pressure inside the voids.

**Additional file 4. Supplementary Fig. S3.** Dynamic surface deformation above a void.

**Additional file 5. Supplementary Table S1.** Water vapor activities of different solutions.

**Additional file 6. Supplementary Fig. S4.** Schematic of the method to derive the location of the void.

**Additional file 7. Supplementary Fig. S5.** Stress–strain curve of the hydrogel.

**Additional file 8. Supplementary Fig. S6.** Simulation results with two different computational domains.

**Additional file 9. Supplementary Fig. S7.** Fabrication of the microfluidic chip.

**Additional file 10. Supplementary Fig. S8.** Depths of the concaves at different spots.
