# Supplementary material for: Non-contact optical characterization of negative pressure in hydrogel voids and microchannels
Source: Front Optoelectron. 2022 Apr 14;15(1):10. doi: 10.1007/s12200-022-00016-5 (PMC9756264; doi:10.1007/s12200-022-00016-5)
Supplement: Supplementary file 1 — Additional file 1. Supplementary Method. Experimental details. [file 12200_2022_16_MOESM1_ESM.pdf]

Experimental details

Fabrication of hydrogel with voids. Figure S1 shows the schematic of the process to fabricate pHEMA hydrogels with micro-voids. pHEMA hydrogel solution consists of 65% [v/v] 2-hydroxyethyl methacrylate (HEMA), 28% [v/v] de-ionized water, 6% [v/v] ethylene glycol dimethacrylate (EGDMA) and 1% [v/v] methacrylic acid (MAA). Then, 1% [v/v] photo-initiator solution (2,2-dimethoxy-2-phenylacetophenone (DMAP) in 1-vinyl-2-pyrrolidone (nVP) with the concentration of 600 mg/mL) was added into the solution. The hydrogel solution was injected into a flask which was previously filled with nitrogen. Then the solution was exposed to UV light (385 nm) for 45 seconds to increase the viscosity. The flask was placed on the vortex mixer (Thermo Fisher Scientific-CN) and vortexed for 45 seconds to introduce bubbles. After mixing, the solution with bubbles was transferred to an acrylic mold through a syringe. The acrylic mold consists of two acrylic slides separated by 1 mm acrylic spacer, and the mold was wrapped by plastic wraps. Finally, the solution was exposed to UV light for about 8 minutes to obtain the pHEMA hydrogel sample with micro-voids. To extract unreacted reagents from the hydrogel and fill the voids with water, the sample was soaked in the boiling de-ionized water for 48 hours and stored in de-ionized water.

Control of water vapor activity. In experiments, we controlled the environmental humidity by different kinds of saturated salt solution. **Table S1** shows the salt solutions used in the experiment and their equilibrium vapor activities. The equilibrium negative pressures calculated through Kelvin-Laplace equation (Eq. (1)) in the main text are also listed. In our experiment, the hydrogel film samples were placed in petri dish, surrounded by cotton filled with saturated salt solution (**Fig. S2**). Then the petri dish was wrapped by Parafilm and placed in Cryostat Water Bath for a week at a constant temperature of 21℃.

Table S1 Water vapor activities of different solutions

Salt	Water vapor activity	Standard deviation	Pressure /MPa	Standard deviation	Reference

K_2SO_4	0.9752	0.0047	−3.31	0.65	[1]
Na_2HPO_4	0.9500	0.0050	−6.87	0.71	[2]
KCl	0.8491	0.0045	−22.15	0.72	[1]

Measurement of size and location of the voids. In the experiment, we measured the size and position of voids through optical microscope (NIKON Instruments Co., Ltd.). After the samples were taken out from the Petri dish, they were wrapped with plastic wraps before observation to avoid the water evaporation. The size or diameter of the void was obtained directly through the microscope measurement. Most voids in the samples were between 50 and 150 μm in diameter. The position or depth of the void in hydrogel film is measured through dynamic focusing of microscope (**Fig. S4**). We adjusted the microscope focusing on the upper, bottom surface of hydrogel film and the center of void sequentially. The vertical movement was recorded to derive the location.

Surface profiling of hydrogel samples. A 3D Optical Surface Profiler (NewView™ 9000**,** Zygo Corporation) was used to characterize the surface deformation. After deriving the size and location, the sample was transferred for surface profile. Here the sample was still wrapped by PE film to avoid evaporation. After we found the target void, we quickly uncovered the plastic wraps and started profiling.

Measurement of Young's modulus of hydrogels. The Young's modulus of the pHEMA hydrogel was tested by In-situ Planer Biaxial Fatigue Testing Systems (IPBF-300**,** CARE Measurement & Control Co.Ltd).

Synthesis of PDMS. The PDMS thick film we used in the experiment is made by mixing the base and the curing agent of Sylgard 184 (Dow Corning) at the weight ratio of 10:1. Then we poured the precursor on the silicon wafer and put it into the vacuum chamber to drive the bubbles out. Finally, the precursor was cured at 80 °C in the oven for 2 hours.

Synthesis of TMSPMA solution. We used 3-(trimethoxysilyl) propyl methacrylate (TMSPMA) solution to modify the solid surfaces so that we could anchor the hydrogels on the surface [3]. We added 3 mL of TMSPMA, 150 mL of deionized water and 20 μL

of acetic acid, then stirred the solution until it became clear.

Fabrication of the microfluidic chip. Figure S7 shows the schematic of the process to fabricate the microfluidic chip. We purchased the glass substrate with designed microchannel from Wuhan Jianmizhikong Technology Co., Ltd, and the PDMS thin films from Shanxi Aiqikang Trading Co., Ltd. Firstly we applied oxygen plasma treatment on both the glass substrate and the PDMS thin film, and then bonded them together immediately after the treatment. A square area on the thin film had been previously cut off to expose the reservoir region of the substrate to the air. To prevent hydrogel breakage during the water filling process, we embedded the hydrogel in the hole of a glass slice. A PDMS thick film was also added to bond the hydrogel slice with the substrate. Finally, we applied oxygen plasma treatment on the surface of thin and thick PDMS films, and then bonded the two parts together. The chip was soaked in deionized water and put in the vacuum chamber to fill it with water.

Supplementary Notes

Comparison of the experiment values with simulation results

Shapes of the surface pit observed in experiments are a little different from the COMSOL simulation results. It might be caused by the mechanical difference of the hydrogel at different water content. It is to say, the hydrogel near the voids may possess larger water content and thus lower Young's Modulus. Hence, in simulation, the results might be inaccurate if we set the whole hydrogel as the same Young's Modulus (**Fig. S6a**). Since the dry pHEMA hydrogel have very high Young's Modulus, here we assumed the low water content pHEMA hydrogel is incompressible, and chose a computational domain covering only the adjacent area of hydrogel void (**Fig. S6b**). As can be seen in **Fig. S6d**, the surface deformation matches very well with the experimental results. Here, the distance between computational domain boundary and void boundary is set as 50 μm. More importantly, the width of the pit varies when we change the computational domain, but the height keeps unchanged. Thus, the fit the height the pit to obtain the negative pressure in the main text.

Calculation of pressure drop along the fluid flow

The theoretical pressure drop along the flow was calculated using the following equation:

$$\Delta P = \frac{128\mu L\varphi}{\rho\pi D^4},$$

where ΔP is the pressure drop, μ is the viscosity of water (taken to be 0.001 kg/(m·s)), L is the length of the channel, φ is the mass flow rate during evaporation, ρ is the density of water (taken to be 1000 kg/m^3), and D is the hydraulic diameter of the channel. Although we observed concave on the film surface during the experiment, the concave had little influence on the hydraulic diameter of the channel. The total pressure drop along the channel was calculated as 0.3056 MPa. Also, we calculated the pressure losses of the laminar flow in 90° channel bends using

$$h = \varepsilon\frac{v^2}{2g},$$

where h is the head loss, ε is the loss coefficient (taken to be 1.1 for 90° bend), v is the velocity, and g is the gravitational acceleration (taken to be 9.8 m/s^2). The total head loss in channel bends was calculated as 0.0001 m (1 Pa), which is negligible compared to the total pressure drop.

Supplementary Figures

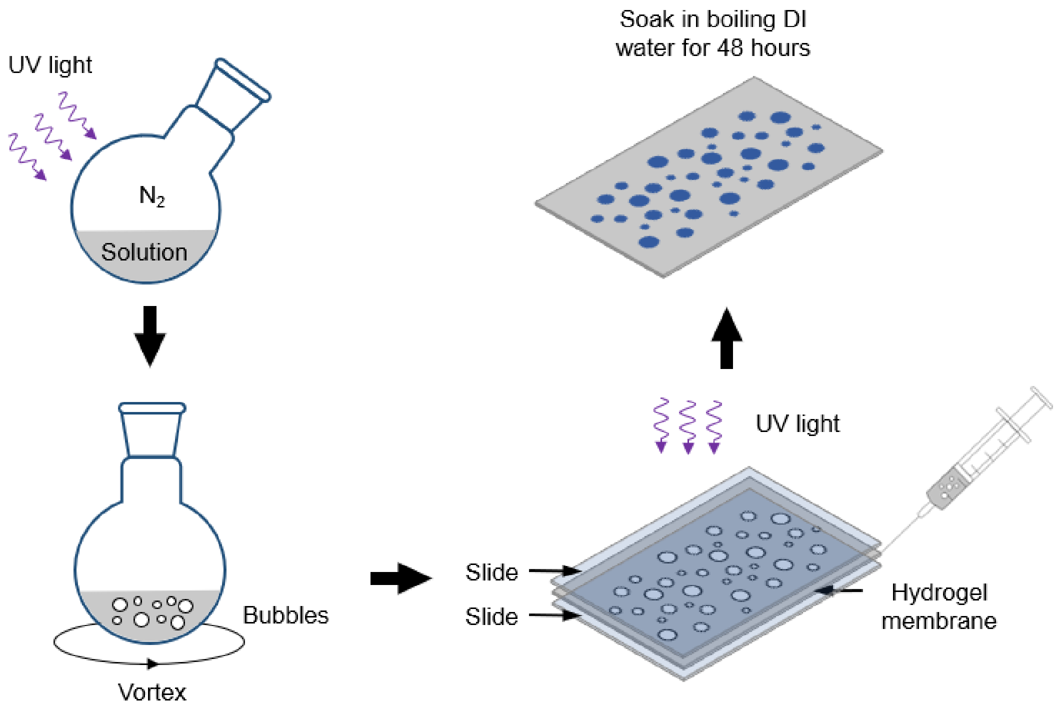

Figure S1. Fabrication of hydrogels with voids. The hydrogel solution was firstly exposed to UV light for a while to increase the viscosity. Then bubbles were introduced into the solution by the vortex mixer. After mixing, the solution with bubbles was immediately transferred to the mold and was exposed to UV light to finish the polymerization. Finally, we filled the voids with water by soaking the hydrogel film in boiling water for 2 days.

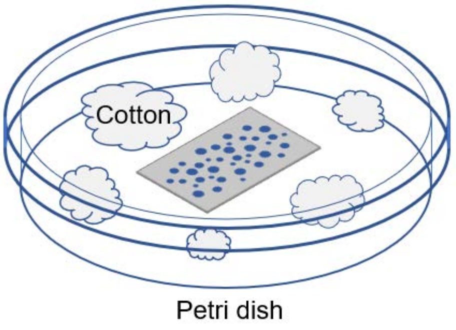

Figure S2. Schematic of the method to generate negative pressure inside the voids. The cotton was firstly soaked in the saturated salt solution, and then placed around the hydrogel sample to control the vapor activity in the Petri dish. The Petri dish was sealed with Parafilm and kept with the constant temperature for a week.

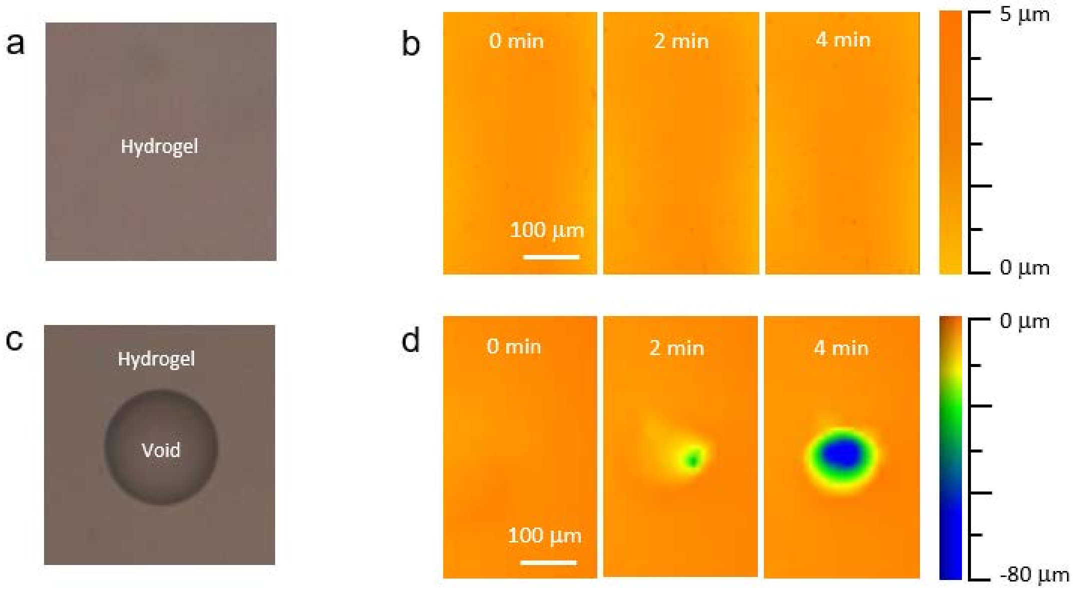

Figure S3. Dynamic surface deformation above a void. (a) Optical photograph of a region without voids in the hydrogel film. (b) Surface deformation of the region without voids during the evaporation. (c) Optical photograph of a void filled with water. (d) Surface deformation of the region during evaporation.

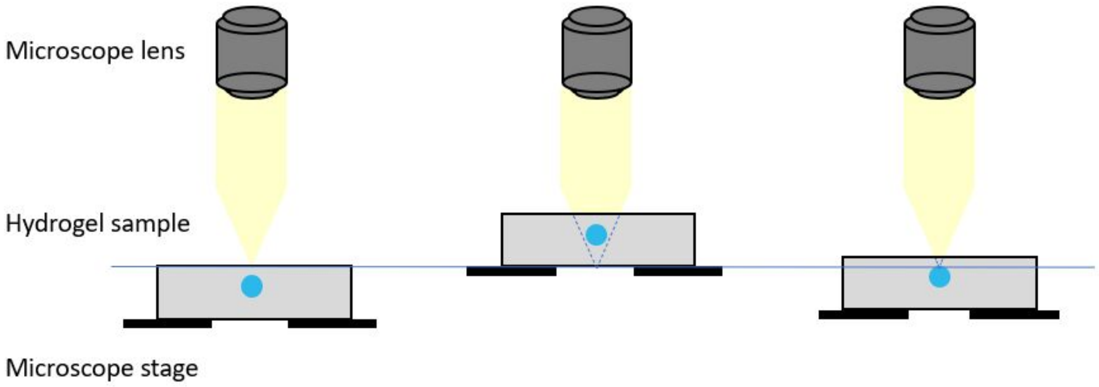

Figure S4. Schematic of the method to derive the location of the void.

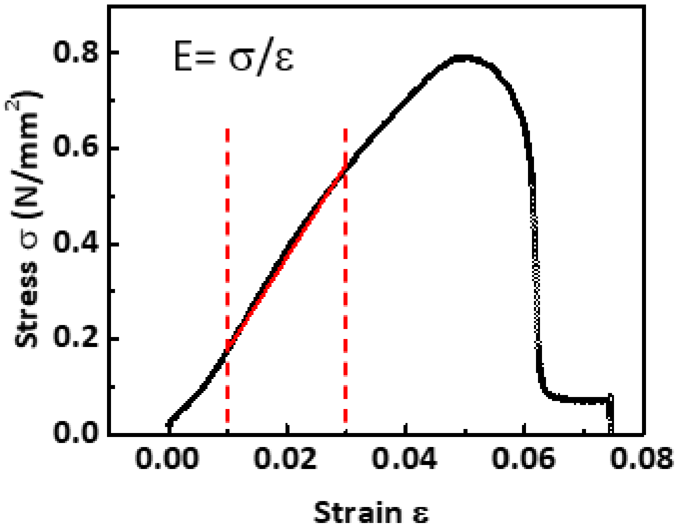

Figure S5. Stress–strain curve of the hydrogel. The linear portion of the curve (marked by red lines) was selected for calculation.

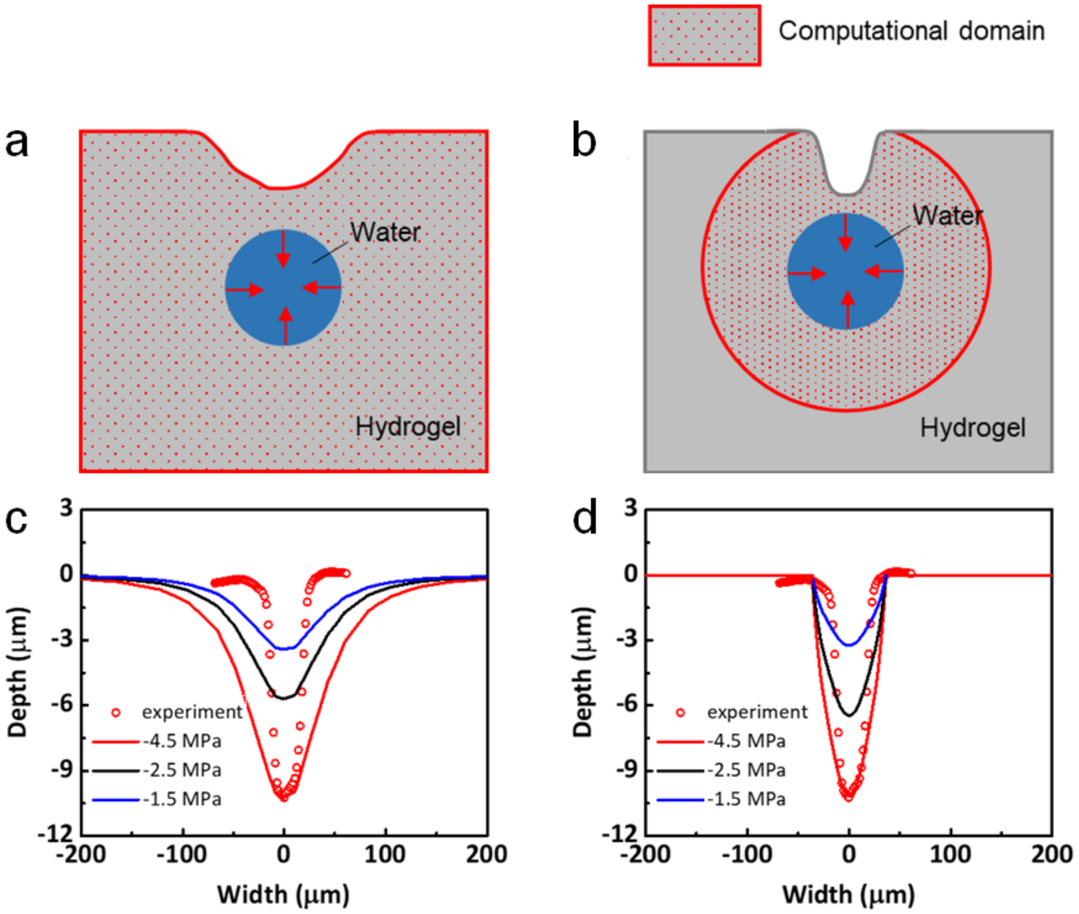

Figure S6. Simulation results with two different computational domains. (a) The whole hydrogel model is set as the computational domain. (b) Only the adjacent area of the void is set as the computational domain. (c) Simulation results with the computational domain as shown in (a). (d) Simulation results with the computational domain as shown in (b).

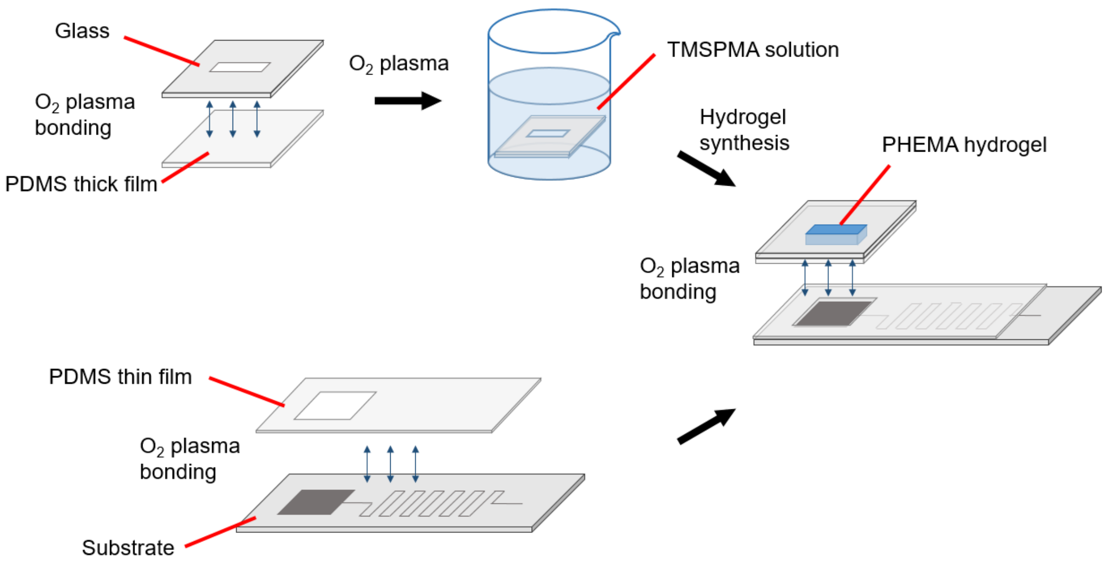

Figure S7. Fabrication of the microfluidic chip. The chip consists of two parts. For the upper part, a piece of glass with hole was bonded with PDMS thick film through oxygen plasma treatment. Then the we modified its surface using TMSPMA solution. After the modification, a piece of pHEMA hydrogel was fabricated in the hole. For the microchannel part, the substrate with channel was bonded with PDMS thin film through oxygen plasma treatment. At last, the two parts were assembled through oxygen plasma treatment.

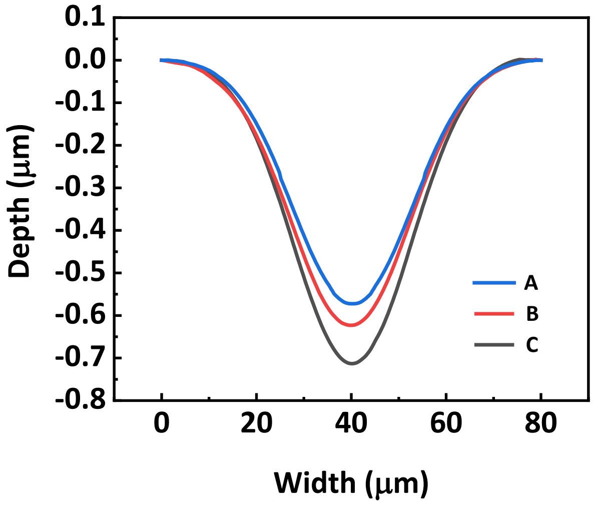

Figure S8. Depths of the concaves at different spots. The depths of concaves at A, B and C are shown in the figure.

References

1. Greenspan, L. Humidity fixed points of binary saturated aqueous solutions. Journal of Research of the National Bureau of Standards A: Physics and Chemistry, 1977, 81A (1): 89–96
2. Winston, P. W. and Bates, D. H. Saturated solutions for the control of humidity in biological research. Ecology, 1960, 41(1): 232–237
3. Yuk H, Zhang T, Lin S, Parada GA, Zhao X. Tough bonding of hydrogels to diverse non-porous surfaces. Nature Materials, 2016, 15(2): 190–196.
