# Supplementary material for: Non-contact optical characterization of negative pressure in hydrogel voids and microchannels
Source: Front Optoelectron. 2022 Apr 14;15(1):10. doi: 10.1007/s12200-022-00016-5 (PMC9756264; doi:10.1007/s12200-022-00016-5)
Supplement: Supplementary file 2 — Additional file 2. Supplementary Fig. S1. Fabrication of hydrogels with voids. [file 12200_2022_16_MOESM2_ESM.pdf]

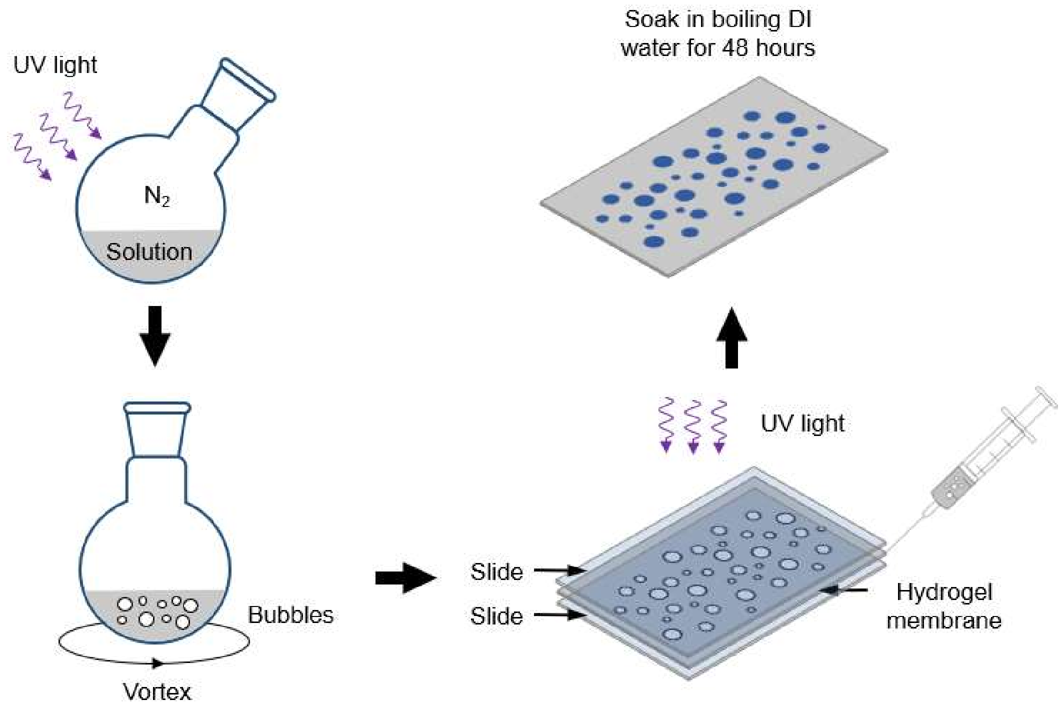

Figure S1. Fabrication of hydrogels with voids. The hydrogel solution was firstly exposed to UV light for a while to increase the viscosity. Then bubbles were introduced into the solution by the vortex mixer. After mixing, the solution with bubbles was immediately transferred to the mold and was exposed to UV light to finish the polymerization. Finally, we filled the voids with water by soaking the hydrogel film in boiling water for 2 days.
