# Supplementary material for: Non-contact optical characterization of negative pressure in hydrogel voids and microchannels
Source: Front Optoelectron. 2022 Apr 14;15(1):10. doi: 10.1007/s12200-022-00016-5 (PMC9756264; doi:10.1007/s12200-022-00016-5)
Supplement: Supplementary file 3 — Additional file 3. Supplementary Fig. S2. Schematic of the method to generate negative pressure inside the voids. [file 12200_2022_16_MOESM3_ESM.pdf]

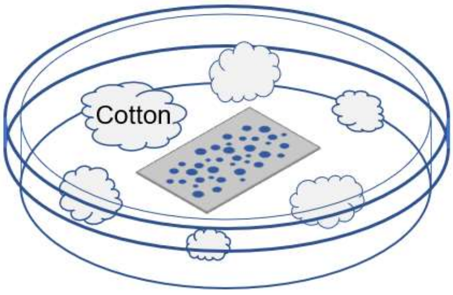

Figure S2. Schematic of the method to generate negative pressure inside the voids. The cotton was firstly soaked in the saturated salt solution, and then placed around the hydrogel sample to control the vapor activity in the Petri dish. The Petri dish was sealed with Parafilm and kept with the constant temperature for a week.
