# Supplementary material for: Non-contact optical characterization of negative pressure in hydrogel voids and microchannels
Source: Front Optoelectron. 2022 Apr 14;15(1):10. doi: 10.1007/s12200-022-00016-5 (PMC9756264; doi:10.1007/s12200-022-00016-5)
Supplement: Supplementary file 4 — Additional file 4. Supplementary Fig. S3. Dynamic surface deformation above a void. [file 12200_2022_16_MOESM4_ESM.pdf]

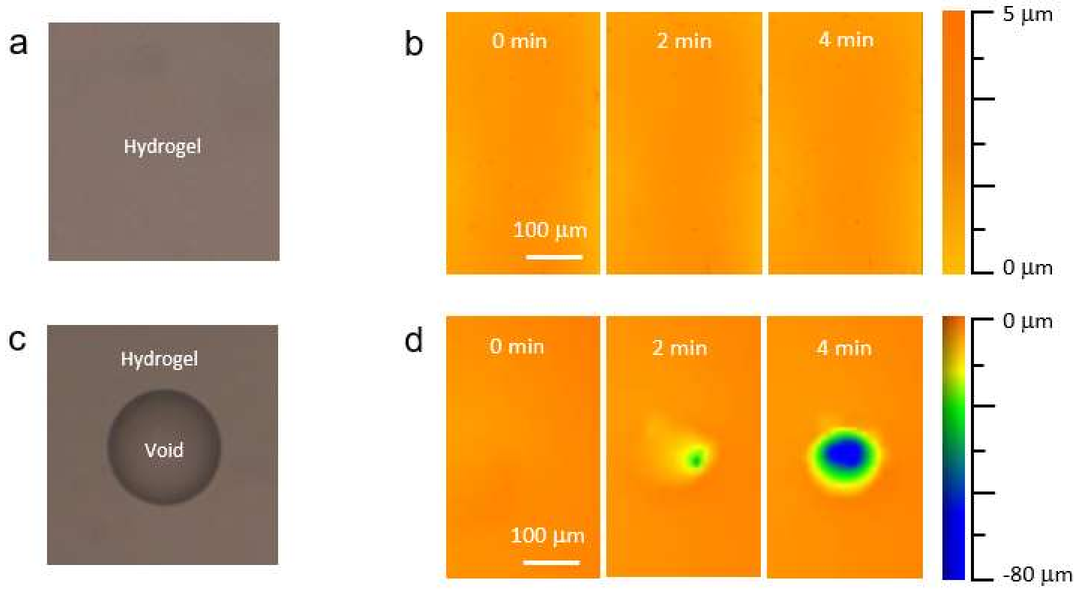

Figure S3. Dynamic surface deformation above a void. (a) Optical photograph of a region without voids in the hydrogel film. (b) Surface deformation of the region without voids during the evaporation. (c) Optical photograph of a void filled with water. (d) Surface deformation of the region during evaporation.
