# Supplementary material for: Non-contact optical characterization of negative pressure in hydrogel voids and microchannels
Source: Front Optoelectron. 2022 Apr 14;15(1):10. doi: 10.1007/s12200-022-00016-5 (PMC9756264; doi:10.1007/s12200-022-00016-5)
Supplement: Supplementary file 5 — Additional file 5. Supplementary Table S1. Water vapor activities of different solutions. [file 12200_2022_16_MOESM5_ESM.pdf]

Table S1 Water vapor activities of different solutions

Salt	Water vapor activity	Standard deviation	Pressure /MPa	Standard deviation	Reference
K_2SO_4	0.9752	0.0047	−3.31	0.65	[1]
Na_2HPO_4	0.9500	0.0050	−6.87	0.71	[2]
KCl	0.8491	0.0045	−22.15	0.72	[1]
