# Supplementary figures and images for: Non-contact optical characterization of negative pressure in hydrogel voids and microchannels
Source: Front Optoelectron. 2022 Apr 14;15(1):10. doi: 10.1007/s12200-022-00016-5 (PMC9756264; doi:10.1007/s12200-022-00016-5)

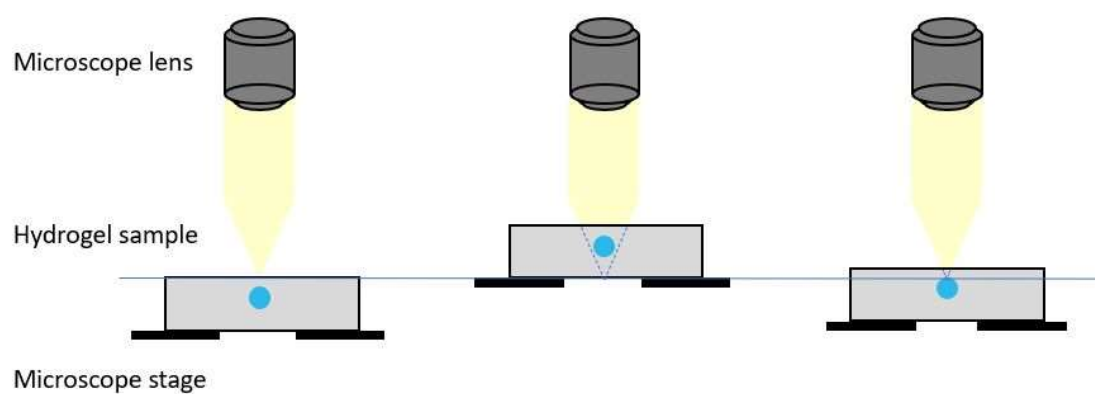

**Figure S4. Schematic of the method to derive the location of the void.**

Supplement: Supplementary file 6 — Additional file 6. Supplementary Fig. S4. Schematic of the method to derive the location of the void. [file 12200_2022_16_MOESM6_ESM.pdf]
