# Supplementary material for: Non-contact optical characterization of negative pressure in hydrogel voids and microchannels
Source: Front Optoelectron. 2022 Apr 14;15(1):10. doi: 10.1007/s12200-022-00016-5 (PMC9756264; doi:10.1007/s12200-022-00016-5)
Supplement: Supplementary file 7 — Additional file 7. Supplementary Fig. S5. Stress–strain curve of the hydrogel. [file 12200_2022_16_MOESM7_ESM.pdf]

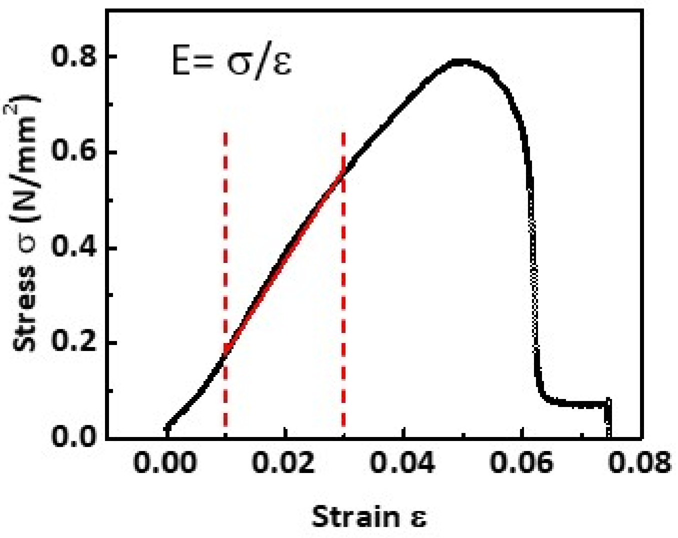

Figure S5. Stress–strain curve of the hydrogel. The linear portion of the curve (marked by red lines) was selected for calculation.
