# Supplementary material for: Non-contact optical characterization of negative pressure in hydrogel voids and microchannels
Source: Front Optoelectron. 2022 Apr 14;15(1):10. doi: 10.1007/s12200-022-00016-5 (PMC9756264; doi:10.1007/s12200-022-00016-5)
Supplement: Supplementary file 8 — Additional file 8. Supplementary Fig. S6. Simulation results with two different computational domains. [file 12200_2022_16_MOESM8_ESM.pdf]

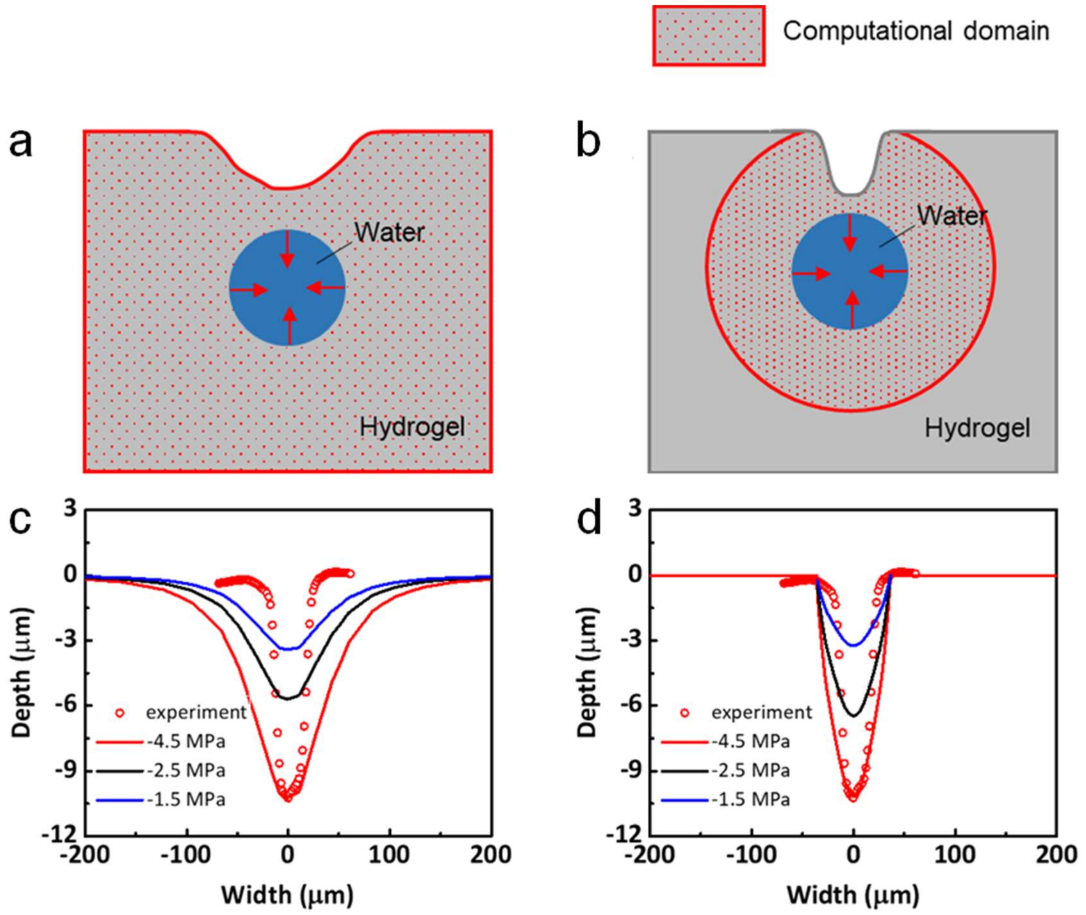

Figure S6. Simulation results with two different computational domains. (a) The whole hydrogel model is set as the computational domain. (b) Only the adjacent area of the void is set as the computational domain. (c) Simulation results with the computational domain as shown in (a). (d) Simulation results with the computational domain as shown in (b).
