# Supplementary material for: Non-contact optical characterization of negative pressure in hydrogel voids and microchannels
Source: Front Optoelectron. 2022 Apr 14;15(1):10. doi: 10.1007/s12200-022-00016-5 (PMC9756264; doi:10.1007/s12200-022-00016-5)
Supplement: Supplementary file 9 — Additional file 9. Supplementary Fig. S7. Fabrication of the microfluidic chip. [file 12200_2022_16_MOESM9_ESM.pdf]

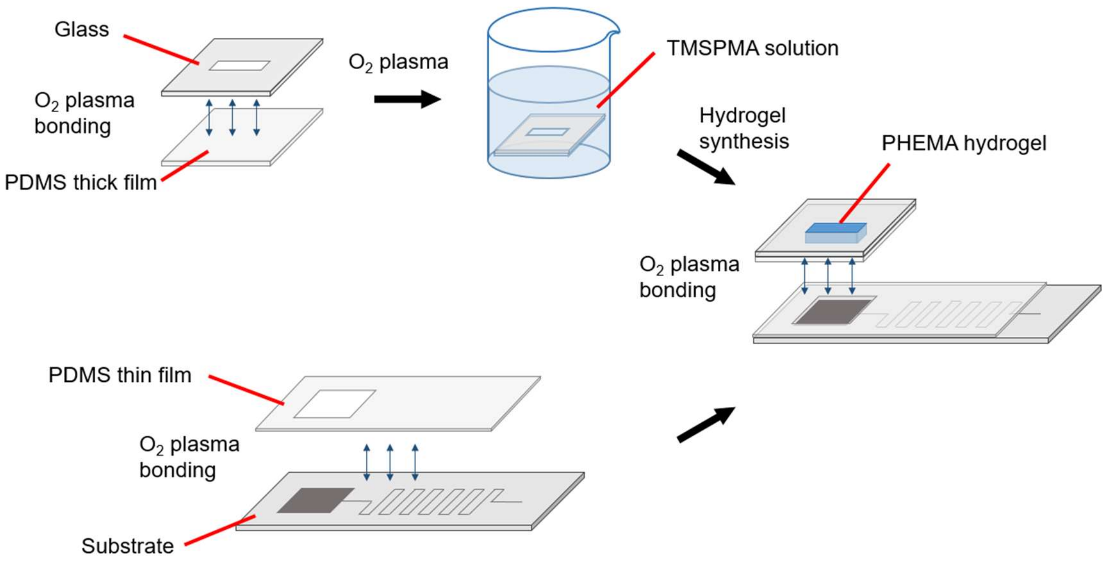

Figure S7. Fabrication of the microfluidic chip. The chip consists of two parts. For the upper part, a piece of glass with hole was bonded with PDMS thick film through oxygen plasma treatment. Then we modified its surface using TMSPMA solution. After the modification, a piece of pHEMA hydrogel was fabricated in the hole. For the microchannel part, the substrate with channel was bonded with PDMS thin film through oxygen plasma treatment. At last, the two parts were assembled through oxygen plasma treatment.
