# Supplementary material for: Non-contact optical characterization of negative pressure in hydrogel voids and microchannels
Source: Front Optoelectron. 2022 Apr 14;15(1):10. doi: 10.1007/s12200-022-00016-5 (PMC9756264; doi:10.1007/s12200-022-00016-5)
Supplement: Supplementary file 10 — Additional file 10. Supplementary Fig. S8. Depths of the concaves at different spots. [file 12200_2022_16_MOESM10_ESM.pdf]

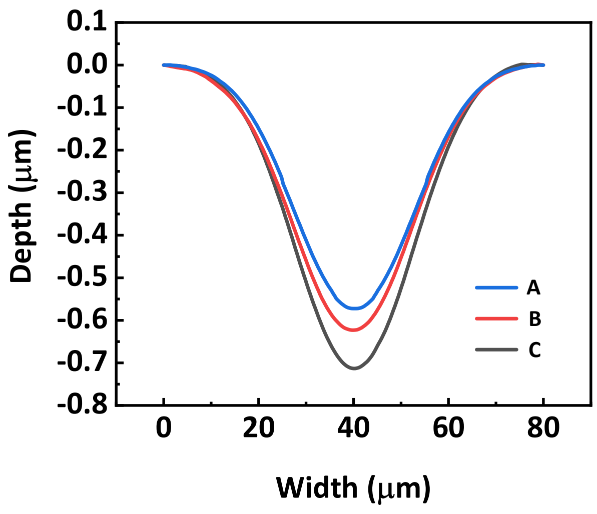

Figure S8. Depths of the concaves at different spots. The depths of concaves at A, B and C are shown in the figure.
